# Design and Fabrication of a Slanted-Beam MEMS Accelerometer

**DOI:** 10.3390/mi8030077

**Published:** 2017-03-07

**Authors:** Wei Xu, Jie Yang, Guofen Xie, Bin Wang, Mingshan Qu, Xuguang Wang, Xianxue Liu, Bin Tang

**Affiliations:** Institute of Electronic Engineering, China Academy of Engineering Physics, Mianyang 621900, Sichuan, China; yjie656@189.cn (J.Y.); xieguofen@126.com (G.X.); wangbinhs@hotmail.com (B.W.); 120214029@qq.com (M.Q.); 554971759@qq.com (X.W.); liuxx1999@163.com (X.L.); john46311@hotmail.com (B.T.)

**Keywords:** MEMS, accelerometer, slanted beam, all-silicon, sandwich structure

## Abstract

This paper presents a novel capacitive microelectromechanical systems (MEMS) accelerometer with slanted supporting beams and all-silicon sandwich structure. Its sensing mechanism is quite similar to an ordinary sandwich-type MEMS accelerometer, except that its proof mass is suspended by a beam parallel to the {111} plane of a (100) silicon wafer. In this way, each sensing element can detect accelerations in two orthogonal directions. Four of these sensing elements could work together and constitute a 3-axis micro-accelerometer by using a simple planar assembly process. This design avoids the traditional 3-axis accelerometer’ disadvantage of possible placement inaccuracy when assembling on three different planes and largely reduces the package volume. The slanted-beam accelerometer’s performance was modeled and analyzed by using both analytical calculations and finite element method (FEM) simulations. A prototype of one sensing element was fabricated and tested. Measured results show that this accelerometer has a good bias stability 76.8 ppm (1σ, tested immediately after power on), two directional sensitivities (sensitivity angle α = 45.4°) and low nonlinearity (<0.5%) over a sensing range up to ±50 g, which demonstrates a great opportunity for future high-precision three-axis inertial measurement.

## 1. Introduction

Microelectromechanical systems (MEMS) accelerometers are used in a wide range of markets and applications and can be classified in several categories: consumer grade, tactical grade and navigation grade depending on their performances, reliability and cost (see Table 1) [1,2]. The consumer grade MEMS accelerometers are quite mature in consumer electronics and automotive electronics markets now [3,4], and are largely driven by lower cost, smaller size and multi-axis integration. On the contrary, the highest inertial navigation-grade markets are still dominated by macro electro-mechanical accelerometers such as Honeywell QA-3000 (Redmond, WA, USA) [5], which can exhibit better than 1 ppm bias composite stability in one year.

With the development of MEMS technologies, MEMS accelerometers are penetrating more and more high-end applications such as aerial navigation, earthquake detection and gravity measurement [6,7,8,9]. Reduction in size of the sensing elements will offer many benefits, but also induce degradation of some specifications, such as measuring resolution, bias stability and the ratio of signal-to-noise. In general, unlike consumer grade accelerometers which are now using surface MEMS + CMOS (complementary metal-oxide-semiconductor) technology for their manufacturing [10], high-precision MEMS accelerometers are still employing bulk silicon micromachining and hybrid assembly technologies [11]. Many successful MEMS manufacturers are providing bulk silicon micro-machined capacitive accelerometers with highly symmetric beam-mass structures, such as Colibrys MS9000 (Neuchatel, Switzerland). Furthermore, the motion measurement of a free body needs to detect accelerations in three orthogonal directions. Common tactical grade MIMUs (Micro Inertial Measurement Units) on the market are orthogonally assembled by three individual and identical single-axis accelerometers on three different planes [12,13]. Thus, these MIMUs are suffering from the disadvantages of bulky package volume and possible assembly misalignment. To overcome these issues, the most effective way is to fabricate all three sensing elements on one single substrate, on the premise of not degrading any performances. There are many different reported approaches to fulfill this goal, such as by using a single proof mass but multiple electrodes for three-axis sensing [14,15], or by placing three sensing elements on a single die [16,17]. The first approach could realize extremely small size devices, but their performances are always limited by high cross-axis sensitivity. Then, the second approach normally uses comb finger sensing structures for in-plane acceleration measurement and sandwich structures for out-of-plane measurement. The combination of two different sensing structures allows a full optimization of each sensing structure’s performance, but it will also result in a much more complex fabrication process. Another possible solution using slanted-beam sensing structures was investigated in this paper, which employs very simple bulk silicon micromachining technologies and can also realize three-axis acceleration detection by fabricating four similar sensing elements on a single die.

In 1995, Andersson et al. firstly fabricated a micro-machined slanted-beam capacitive accelerometer with an asymmetrical structure [18]. Later, in 2005, they extensively studied its working theory and performed device characterization at the Imego institute [19,20]. Because these accelerometers did not use differential detecting principles, their measured performances were quite limited. In addition, S. Butefisch et al. have also fabricated a glass-silicon-glass sandwich structure slanted-beam accelerometer aimed for a low-g sensor application [21]. This sensing structure seems to be symmetrical, but because of the undercutting of silicon sidewalls during KOH etching, the fabricated proof mass can hardly ensure good symmetry. In capacitive accelerometers, it has been evidently demonstrated that accelerometers with highly symmetrical structures can exhibit much better performances [7,8,9].

In this paper, a fully symmetrical slanted-beam accelerometer with all-silicon sandwich structures is presented. Its high symmetry is ensured by a specifically developed wet etch technique, and its all–silicon homogeneous structure can avoid the shortcomings of thermal coefficient mismatch and bring in chip-scale hermetic sealing. The fabricated prototype exhibits a good bias stability and low nonlinearity, experimentally demonstrating the effectiveness of our proposed design and fabrication technology.

## 2. Working Principles

**Figure 1:**
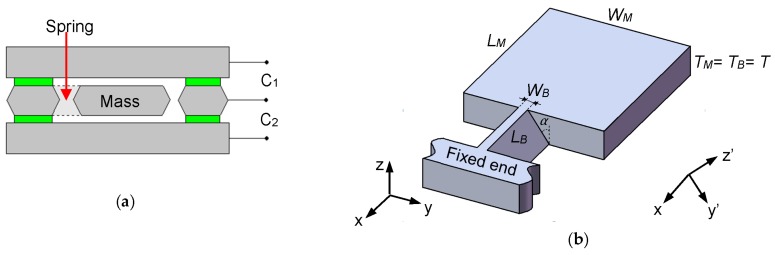
(**a**) Cross-sectional view of the proposed microelectromechanical systems (MEMS) accelerometer; (**b**) 3D drawing of a single slanted-beam sensing element with two different coordinate systems.

The working principles of this proposed accelerometer is based on a traditional differential capacitance detecting system, as shown in Figure 1a. Its MEMS sensing part is composed of three different silicon layers, and the middle layer consists of a proof mass and a support spring that serve as the inertial sensing elements. When acceleration is applied, the proof mass will move upward or downward and cause a capacitance change (Δ*C* = *C*_1_ − *C*_2_) that could be detected by the readout circuits. Then, the readout circuits will produce a voltage, and the change of this output voltage is proportional to applied acceleration.

The main difference of this slanted-beam accelerometer compared to ordinary spring-mass sensing structure is that its suspended beam is parallel to the {111} plane of a (100) silicon wafer (shown in Figure 1b). Thus, its most sensitive direction is inclined to the wafer surface by an angle α = 35.3°, which is normally given by the crystal orientation of the monocrystalline silicon.

In order to simplify the analytical modeling, besides the normal coordinate system (*x*, *y*, *z*), another local coordinate system (*x*, *y’, z’*) is established with the *z*’-axis aligned normally to {111} the plane of the silicon crystal. 

When an acceleration in the *z’*-direction (*a_z‘_*) is applied on the sensing structure, the displacement and rotation of the outer tip of the supporting beam can be calculated by using the beam deflection theory [22]. Its maximum displacement in the *z’* direction is:

(1) $$d_{\max}=\frac{F\times \left(4L_{B}^{3}+3L_{B}^{2}L_{M}\right)}{12EI}.$$

The rotation angle of the proof mass around the *y’*-axis is:

(2) $$\theta =\frac{F\times L_{B}\times \left(L_{B}+L_{M}\right)}{2EI}.$$

Here, geometrical parameters are outlined in Figure 1b, *E* is the Young’s modulus of silicon in (110) direction, and *I* is the moment of inertia of the slanted beam:

(3) $$I=\frac{{\left(W_{B}\cos\alpha \right)}^{3}\times \left(T/\cos\alpha \right)}{12}=\frac{W_{B}^{3}T\cos\alpha^{2}}{12}.$$

*F* is the inertial force generated on the center of the proof mass:

(4) $$F=ma_{z^{\prime }}=\rho L_{M}W_{M}Ta_{z^{.}}$$

Here, ρ is the density of silicon. Because capacitors are formed between two adjacent silicon wafers, only displacement of the proof mass in the *z*-direction will bring changes in their capacitance values. When an acceleration in the *z’*-direction is applied, the *z*-axis displacement at any point of the proof mass (location is defined as *x*) can be obtained as follows:

(5) $$d_{z,z\prime }\left(x\right)=d_{\max}\cdot \sin\alpha +\theta \cdot x\cdot \sin\alpha =\frac{12\sin\alpha }{\cos\alpha^{2}}\cdot \frac{\rho L_{M}W_{M}L_{B}a_{z^{\prime }}}{EW_{B}^{3}}\cdot \left(\frac{L_{B}^{2}}{3}+\frac{L_{B}L_{M}}{4}+\frac{xL_{B}}{2}+\frac{xL_{M}}{2}\right).$$

The calculation of sensitivity in the beam‘s stiffer direction, the *y’*-axis, is analogous to the derivation process of Equation (5). The relationship between *d_z,z’_(x)* and *d_z,y’_(x)* is shown as below:

(6) $$\frac{d_{z,z\prime }}{d_{z,y\prime }}=\frac{\sin\alpha \cdot T^{2}}{\cos^{5}\alpha \cdot W_{B}^{2}}.$$

A readout circuit is used to detect the capacitances, and its output voltage is given by:

(7) $$V_{\mathrm{out}}=k\frac{C_{1}-C_{2}}{C_{1}+C_{2}}.$$

Here, *k* is the total gain of the signal amplifier. Thus, the overall sensitivity of the accelerometer is:

(8) $$S=\frac{V_{\mathrm{out}}}{a}=\frac{k}{a}\cdot \frac{C_{1}-C_{2}}{C_{1}+C_{2}}\approx \frac{k}{a}\cdot \frac{\frac{\varepsilon A_{0}}{d_{0}-\Delta d}-\frac{\varepsilon A_{0}}{d_{0}+\Delta d}}{\frac{\varepsilon A_{0}}{d_{0}-\Delta d}+\frac{\varepsilon A_{0}}{d_{0}+\Delta d}}=\frac{k}{a}\cdot \frac{\Delta d}{d_{0}}.$$

Here, *a* is the applied acceleration, *A*_0_ is the plate area of each capacitor, *d*_0_ is the original gap distance, *Δd* is the average *z*-axis displacement of the proof mass, and *ε* is the permittivity of the gas in the gap. The sensitivity angle of the slanted-beam sensing structure can be calculated as follows:

(9) $$\tan\alpha_{r}=\frac{S_{z}}{S_{y}}=\frac{d_{z,z}}{d_{z,y}}=\frac{\sin\alpha \cdot d_{z,z^{\prime }}+\cos\alpha \cdot d_{z,y^{\prime }}}{\cos\alpha \cdot d_{z,z^{\prime }}+\sin\alpha \cdot d_{z,y^{\prime }}},\;\alpha_{r}\approx \arctan\left(\frac{1}{\sqrt{2}}\right)+\arctan\left(\frac{4\sqrt{2}}{9}{\left(\frac{W_{B}}{T_{M}}\right)}^{2}\right).$$

Here, *S_y_* and *S_z_* represent the sensitivities of the slanted-beam accelerometer in the *y*- and *z*-directions, respectively.

**Figure 2:**
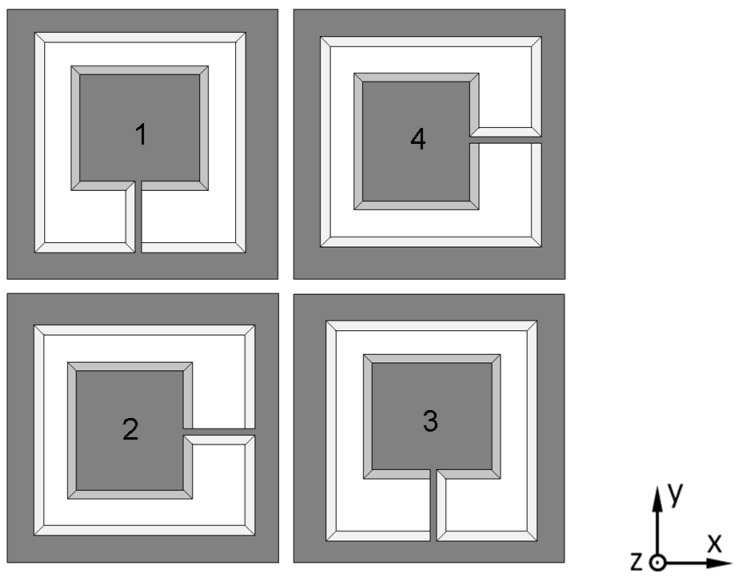
Arrangement of a three-axis MEMS accelerometer that consists of four slanted-beam sensing elements.

After analyzing the performance of one sensing structure, a three-axis design of the MEMS accelerometer is constituted by four of these sensing elements. As shown in Figure 2, all of these sensing elements have the same geometrical parameters, and each one’s location is rotated by 90° in the arrangement.

Because each sensing element can detect accelerations in two orthogonal directions, four output signals related to three-axis accelerations could be obtained:

Out_1_ = *S*_1,*x*_·*a_x_* − *S*_1,*z*_·*a_z_*, Out_2_ = *S*_2,*y*_·*a_y_* − *S*_2,*z*_·*a_z_*, Out_3_ = −*S*_3,*x*_·*a_x_* − *S*_3,*z*_·*a_z_*, Out_4_ = −*S*_4,*y*_·*a_y_* − *S*_4,*z*_·*a_z_*. (10)

Here, *S_i,j_* are the sensitivities in the *j*-direction of sensing element *i*, and *a_j_* are the accelerations in the *j*-direction. Then, a simple calculation needs to be performed after each measurement to separate three individual accelerations along the *x*-, *y*- and *z*-directions:

(11) $$a_{x}=\frac{{\mathrm{Out}}_{1}S_{3,z}-{\mathrm{Out}}_{3}S_{1,z}}{S_{1,x}S_{3,z}+S_{1,z}S_{3,x}},\;a_{y}=\frac{{\mathrm{Out}}_{2}S_{4,z}-{\mathrm{Out}}_{4}S_{2,z}}{S_{2,y}S_{4,z}+S_{2,z}S_{4,y}},\;a_{z}=\frac{-{\mathrm{Out}}_{1}S_{3,x}-{\mathrm{Out}}_{3}S_{1,x}}{S_{1,z}S_{3,x}+S_{1,x}S_{3,z}}.$$

## 3. Simulation and Structural Analysis

**Figure 3:**
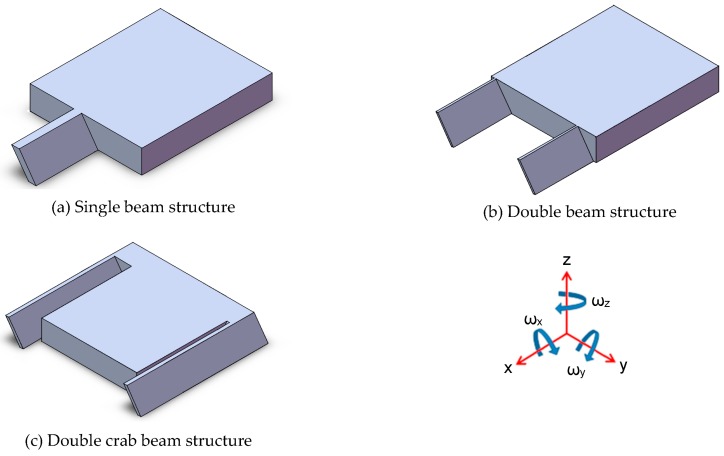
Three different simplified slanted beam-mass structures considered for analysis: (**a**) single beam structure; (**b**) double beam structure; (**c**) double crab beam structure.

In the following content, three different simplified slanted beam-mass structures, as shown in Figure 3, were analyzed by using a Finite Element Modeling (FEM) method and compared for their performances.

As they are not conventional spring-mass structures, potential rotation of the proof mass is a major concern at the initial design step. As shown in Figure 3, ω_*z*_ rotation will not change the capacitance value, and ω_*y*_ rotation is a normal swing similar to the behavior of a traditional cantilever-type accelerometer; hence, only ω_*x*_ rotation is an unwanted movement. A comparison of these three structures, regarding their rotations under different directional applied accelerations, was performed. Geometrical parameters of our proposed design are given in Table 2, and the comparative results are listed in Table 3. What needs to be mentioned is that different dimensions of the slanted beams are used in this analysis in order to obtain similar output signal, which is given by the differential capacitance ratio: (*C*_1_ − *C*_2_)/(*C*_1_ + *C*_2_). As shown in Table 3, all of these three structures exhibit extreme small ω_*x*_ rotation (ω_*x*_ ≤ 1.4 × 10^−5^) when applied with 50 g acceleration, thus proving themselves good candidates for this inertial measurement.

Then, among them, a single beam mass structure was chosen for further investigation because of its geometrical simplicity, lower spring stiffness (considered for the same dimensions of the slanted beams) and internal stress relief of the whole beam mass structure.

Both analytical calculations and Finite Element Modeling (FEM) were performed to analyze this sensing element’s performance. The comparative results are shown in Table 4. Because the two parallel plate method has neglected the capacitance fringe effects, the analytical calculation method gives a slight smaller value of initial capacitance value (*C*_0_ = 16.7 pF) than the FEM method (*C*_0_ = 16.8 pF). When a 50 g acceleration normal to the {111} plane is applied on the beam mass structure, the proof mass will move 0.136–0.667 μm in the *z*-direction, which brings in a capacitance change of 7.15 pF and a differential ratio of 0.202. Then, the device’s sensitivity angle was calculated by using both Equation (9) and FEM methods, and the two results are, as expected, both larger than the silicon crystal orientation 35.3°. The dynamic analysis of this sensing element was also performed. The first resonance mode of this structure corresponds to a vibration normal to the {111} plane of the slanted supporting beam, and its resonance frequency is 3.9 kHz. The second resonance mode corresponds to the undesired roll rotation with a resonance frequency of 16 kHz. Since the natural frequency of the first mode is much smaller than higher modes, the sensor’s sensing mode proves to be successfully decoupled from other unwanted vibration modes.

**Figure 4:**
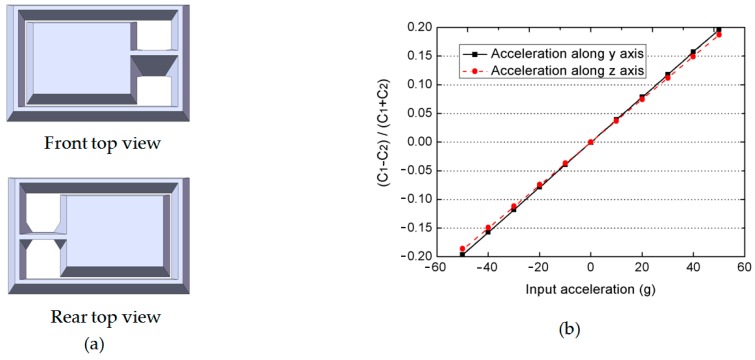
(**a**) A more accurate 3D model used for simulation; (**b**) simulated output signals (differential capacitance ratio: (*C*_1_ − *C*_2_)/(*C*_1_ + *C*_2_)) as a function of applied accelerations in two orthogonal directions.

Since former analysis is only based on simplified models, a more accurate 3D model is established to simulate the performance of the real fabricated devices. As shown in Figure 4a, convex corners will be formed at both ends of the slanted supporting beam because of the etching properties of the chosen wet etchant.

Simulated output signals (differential capacitance ratio: (*C*_1_ − *C*_2_)/(*C*_1_ + *C*_2_)) as a function of applied accelerations in both the y- and z-axes are plotted in Figure 4b. These two lines show very good linearity (nonlinearity: 0.35‰ on the *y*-axis and 0.45% on the *z*-axis) over a sensing range up to ±50 g, and the sensitivities in two orthogonal directions only have a very small difference (*k*_*y*_ = 3.93 × 10^−3^, *k*_*z*_ = 3.75 × 10^−3^). Its sensitive angle could then be calculated to be 43.7°.

Another issue about this accelerometer is the influence of its parasitic capacitance. Since it has a rigid outer frame (frame width = 50 am) to a protect central sensitive area, large parasitic capacitance (≈10.5 pF) is generated from the bonding SiO_2_ area and may worsen the accelerometer’s nonlinearity performance. This effect is eliminated by the readout circuit design. Actually, a more detailed transfer function of the detecting circuit is expressed as:

(12) $$V_{\mathrm{out}}=k\frac{\left(C_{1}+C_{p1})-(C_{2}+C_{p2}\right)}{(C_{1}+C_{p1})+(C_{2}+C_{p2})-C_{\mathrm{comp}}}.$$

Here, *C*_1_ and *C*_2_ are variable capacitances of the upper and lower capacitor, *C_pi_* are their parasitic capacitances, *C*_comp_ is a programmable compensation capacitance integrated in the front end C-V converter. This slanted-beam accelerometer has a very good symmetry to ensure *C_p_*_1_ = *C_p_*_2_. Then, by adjusting the programmable compensation capacitance *C*_comp_, the sum of *C_p_*_1_ and *C_p_*_2_ can be subtracted in this transfer function.

## 4. Device Fabrication

**Figure 5:**
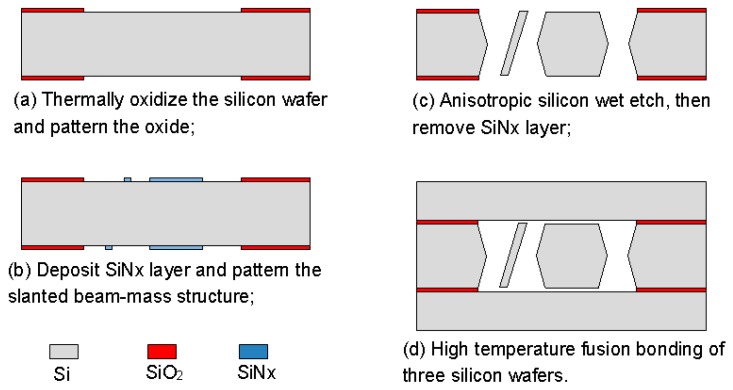
A brief description of the proposed fabrication process.

The proposed fabrication process flow of the single slanted-beam accelerometer is briefly outlined in Figure 5. Firstly, a double side polished silicon wafer (4 inch, thickness 380 μm, (100) orientation, resistivity 0.1 Ω·cm) is thermally oxidized to grow 2 μm SiO_2_ on its two surfaces at 1100 °C. Subsequently, the two oxide layers are patterned by using double-sided mask aligner and BHF (Buffered Hydrofluoric Acid) etch (Figure 5a). These oxide layers serve as electrical isolation and define the initial distance of capacitance gap. Next, the whole silicon wafer is deposited by a 600 nm SiN*x* layer on both of its sides (Figure 5b), and these SiN*x* layers are patterned to form the etching mask of the slanted beam-mass sensing structure. The silicon wafer is then etched in wet anisotropic etching solution until the slanted beam is successfully formed (Figure 5c). This structured silicon wafer and two other silicon wafers are hermitically sealed by using a high temperature Silicon Fusion Bonding (SFB) process (Figure 5d). Finally, a film of Cr/Au is simultaneously evaporated on three different layers to define the contacts’ metallization.

The wet etch process is performed with 25 wt % TMAH (Tetramethylammonium Hydroxide) + 0.1% (*v*/*v*) Triton-X-100 [C_14_H_22_O(C_2_H_4_O)_*n*_, *n* = 9–10] solution. A constant temperature bath at 80 °C is used to keep the etchant temperature within ±1 °C. Our formerly reported results [7] have already demonstrated that additive-modified TMAH could provide the minimum undercutting at the mass corner. However, this time, the wet etch process needs a much longer time than etching through the wafer. According to our experiences, the effect of additive has already reached its limit and can no longer be competent for this task. Thus, a specific wet etching technique is developed mainly based on designing a compensation pattern in the masking layer and combining it with an additive-modified solution. Different stages of the technical development and their corresponding fabricated results are shown in Table 5. As we can see, after adding both the additive solvent and a ‘T’ shape compensation structure (*d* = 10 μm, *l* = 40 μm), a well protected convex corner is successfully fabricated.

**Figure 6:**
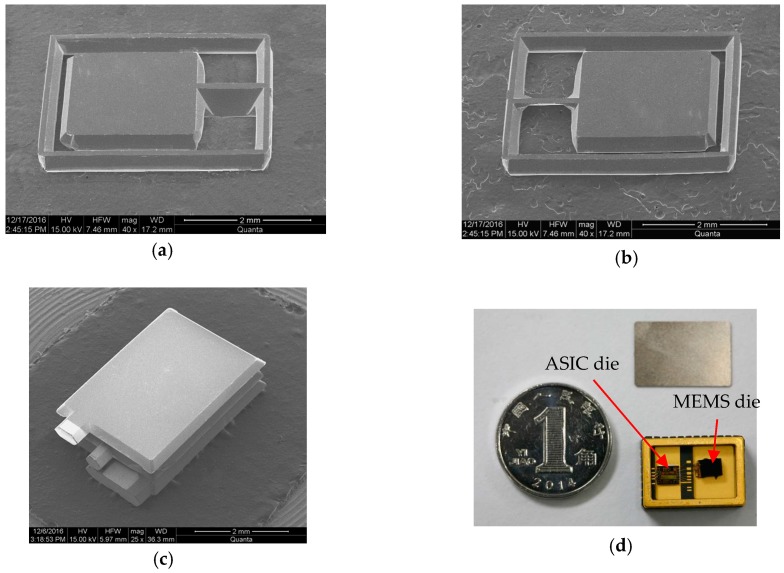
(**a**) front side and (**b**) back side SEM images of the fabricated single slanted beam mass structure; (**c**) SEM images of the bonded three-layer device; (**d**) Leadless Chip Carriers (LCC) package including the MEMS and Application Specific Integrated Circuit (ASIC).

Then, this middle silicon wafer is bonded with two other silicon wafers to form the differential capacitors. Care must be taken about the cleaning of the bonding surface prior to the temporal Si-SiO_2_ bonding. A high temperature annealing (>1000 °C) for 2 h is needed to permanently seal the cavity. After these process steps, a slanted-beam MEMS die with a highly symmetrical spring-mass structure has been fabricated. Figure 6a,b show the front side and back side SEM images of fabricated single slanted beam-mass structure, and Figure 6c shows the bonded three-layer device. Thereafter, the MEMS die is diced and assembled with a proprietary Application Specific Integrated Circuit (ASIC) die in a hermetically sealed Multichip Module (MCM) as shown in Figure 6d.

## 5. Measurement Results

**Figure 7:**
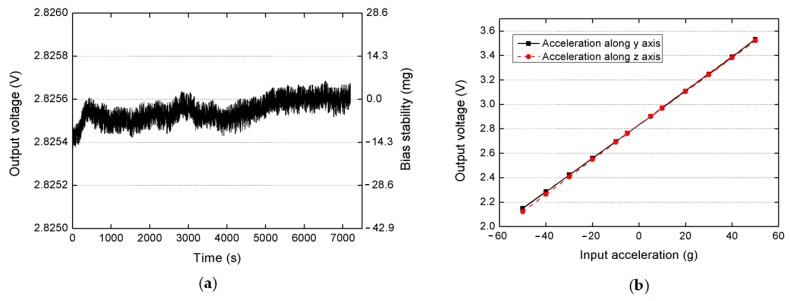
(**a**) bias drift of a packaged slanted-beam accelerometer; (**b**) centrifugal measurement of a packaged device in the range of ±50 g.

A preliminary test was performed to validate the effectiveness of a fabricated slanted-beam accelerometer. The bias stability performance was measured at room temperature for 12 min without any compensation. Its output signal was recorded immediately after the power was turned on, and the result in Figure 7a shows that this accelerometer has demonstrated a short-time bias stability of 3.84 mg (1σ) or 15.7 mg (maximum drift). The white noise of this accelerometer was also measured to be 40.2 μV/√Hz. Then, the accelerometer’s dynamic response was also measured by using a precise centrifuge in the range of ±50 g. The plots in Figure 7b show that the accelerometer almost has a similar scale factor in two orthogonal directions (13.79 mV/g on the *y*-axis and 13.98 mV/g on the *z*-axis), and its nonlinearity in two directions are both smaller than 0.5% (0.48% on the *y*-axis and 0.49% on the *z*-axis). The sensitivity angle is calculated to be 45.4°, which is larger than simulated value 43.7°. This difference results from the geometrical errors of fabricated slanted beams and could be minimized by future optimization of the fabrication processes. A comparison of FEM values and measured values of this prototype are listed in Table 6.

Table 7 shows the comparison of some recently reported capacitive MEMS accelerometers with the slanted-beam accelerometer fabricated in this work. It can be seen that this accelerometer needs very simple bulk silicon fabrication processes, while also achieving good performance. These measured results demonstrate the effectiveness of our proposed design and fabrication technology. Since this prototype only used open loop control ASIC, a close loop circuit is under consideration to achieve better nonlinearity and lower noise.

## 6. Conclusions

In this paper, a slanted-beam MEMS capacitive accelerometer based on a highly symmetrical all-silicon sandwich structure is present. Three different beam-mass structures were firstly analyzed and compared for their performances. Then, among them, a single beam sensing structure was chosen for further investigation because of its geometrical simplicity, lower spring stiffness and internal stress relief of the whole beam mass structure. Both analytical calculation and FEM simulation were performed to analyze the sensor’s performance. A simple fabrication process mainly based on bulk silicon fabrication technologies is also presented. Since slanted beams need much longer etching time than etching through the wafer, a specific wet etch technique modified by both additive solvent and geometrical compensation patterns was developed for this task. Finally, a highly symmetrical slanted-beam structure was successfully fabricated and assembled with a proprietary ASIC die for device characterization. The measured results prove that this accelerometer has a good bias stability (76.8 ppm, 1σ), two directional sensitivities (sensitivity angle α = 45.4°) and low nonlinearity (<0.5%) over a sensing range up to ±50 g. Future work is to integrate four slanted-beam accelerometers on a single plane and to realize three-axis inertial measurement.

## Figures and Tables

**Table 1:** The categories of microelectromechanical systems (MEMS) accelerometers [1,2].

Performance	Consumer Grade MEMS	Tactical Grade MEMS		Navigation Grade Electromechiancal
		Open Loop	Close Loop	
Bias stability	1000 ppm	100 ppm	10 ppm	1 ppm
Signal-to-noise	80 dB	100 dB	120 dB	140 dB

**Table 2:** Geometrical parameters of our proposed single slanted-beam sensing element.

Parameter	Symbol	Value
Mass thickness	*T_M_*	380 μm
Mass length	*L_M_*	2100 μm
Mass width	*W_M_*	1800 μm
Slanted-beam thickness	*T_B_*	380 μm
Slanted-beam length	*L_B_*	1000 μm
Slanted-beam width	*W_B_*	120 μm
Air gap	*g*	2 μm

**Table 3:** Output signals and rotations of the proof mass under different directional applied accelerations.

Different Structures	Applied Accelerations	*C*_1_ − *C*_2_ (pF)	$\frac{C_{1}-C_{2}}{C_{1}+C_{2}}$	ω_*x*_ (°)	ω_*y*_ (°)	ω_*z*_ (°)	Dimensions of Slanted Beam
Single beam structure	*a_x_* = 50 g	~0	0	5.7 × 10^−6^	3.1 × 10^−9^	2.4 × 10^−7^	*L_B_, W_B_*, *T_B_*
	*a_y_* = 50 g	5.31	0.153	8.4 × 10^−8^	1.1 × 10^−2^	1.6 × 10^−2^	
	*a_z_* = 50 g	4.52	0.131	3 × 10^−8^	9.5 × 10^−3^	1.1 × 10^−2^	
Double beam structure	*a_x_* = 50 g	~0	0	1.7 × 10^−6^	7.3 × 10^−8^	2 × 10^−7^	*L_B_*, $\frac{1}{4}$ *W_B_*, *T_B_*
	*a_y_* = 50 g	4.54	0.132	7.7 × 10^−7^	7.6 × 10^−5^	8.4 × 10^−5^	
	*a_z_* = 50 g	4.78	0.139	1.6 × 10^−6^	3.3 × 10^−3^	5.7 × 10^−5^	
Double crab beam structure	*a_x_* = 50 g	~0	~ 0	1.4 × 10^−5^	1.4 × 10^−7^	1.7 × 10^−6^	*2L_B_*, $\frac{1}{2}$ *W_B_*, *T_B_*
	*a_y_* = 50 g	6	0.173	1 × 10^−6^	9.9 × 10^−5^	2 × 10^−5^	
	*a_z_* = 50 g	4.55	0.133	2.6 × 10^−7^	7.7 × 10^−4^	6.7 × 10^−6^	

**Table 4:** Theoretically predicted performance of this device.

Device Performance		Analytical Value	FEM Value
Initial capacitance value (*C*_0_ = *C*_1_ = *C*_2_)		16.7 pF	16.8 pF
Apply 50 g acceleration normal to {111} plane	Maximum *z*-axis displacement of the proof mass	0.632 μm	0.667 μm
	Minimum *z*-axis displacement of the proof mass	0.132 μm	0.136 μm
	Differential capacitance *C*_1_ − *C*_2_	6.4 pF	7.15 pF
	Differential ratio (*C*_1_ − *C*_2_)/(*C*_1_ + *C*_2_)	0.191	0.202
Sensitivity angle		38.9°	40.4°
Resonance frequency		-	*f*_0_ = 3.9 kHz *f*_1_ = 16 kHz *f*_2_ = 18.4 kHz

**Table 5:** Different stages of the developed wet etch technique and their fabricated results.

Different Stages of Developed Wet Etch Technique	Etch Time	Fabricated Result
1. Additive modified TMAH	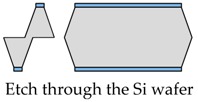	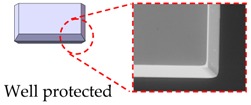
2. Additive modified TMAH	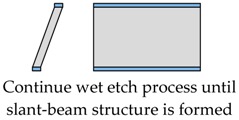	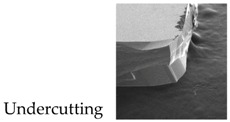
3. Additive modified TMAH + compensation structure	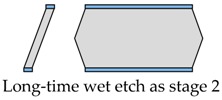	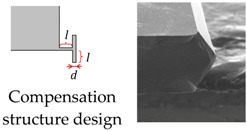

**Table 6:** Comparison of finite element method (FEM) values and measured values of the fabricated accelerometer.

Device Performance	FEM Value	Measured Value
Sensitivity angle	43.7°	45.4°
Non linearity on the *y*-axis	0.35‰	0.48%
Non linearity on the *z*-axis	0.45‰	0.49%

**Table 7:** Performance comparison of fabricated accelerometer with other capacitive accelerometers reported recently.

Different Accelerometers	Ref. [14] 2013	Ref. [16] 2015	Ref. [9] 2016	This Work
Type	Three-axis accelerometer Single mass + multiple electrodes	Three-axis accelerometer Single mass + multiple electrodes	Single-axis accelerometer Sandwich structure	Slanted beam structures
Full range	3 g	10 g	1 g	50 g
Bias stability ^1^	-	200 ppm (lateral) 3500 ppm (vertical)	1000 ppm 70 ppm (20 min later)	314 ppm 76.8 ppm (1σ)
Non linearity	2.57%–2.91%	0.28%–0.41%	0.66%	0.49%

Bias stability ^1^: Maximum drift of the bias immediately measured after power on. The value listed in the table is the ratio of measured bias stability to full sensing range, thus given in ppm.
